# Letter to Editor Regarding “Involving the Family in Patient Care: A Culturally Tailored Communication Model” by Jazieh *et al.*

**DOI:** 10.4103/JQSH.JQSH_2_20

**Published:** 2020-02-14

**Authors:** Eman Kamal

**Affiliations:** Department of Quality Management and Patient Safety Administration, King Saud Medical City, Riyadh, Saudi Arabia

Dear Editor,

The fact that the article titled “Involving the family in patient care: A culturally tailored communication model,”[1] is contextualized to an Arab culture where the number of family members is large intrigued me to study the subject. I found the abstract to be highly informative, motivating me to read about the model and process of family involvement. I have read through the entire content and realized that interacting effectively with large families is a challenging task in terms of communication because decision-making in larger families is difficult to manage.

In general, physicians play a vital role as the center of contact in a hospital setup. The beneficiaries are known to be patients and families. A text can be viewed as a connection between the doctor and the relationships of the patient. Different perception by both parties of the message suggests a positive system of interaction.[2]

In a background of physician–relatives combined patient care, a sequence of interactions may occur between the physicians and relatives, ultimately resulting in a summated effect.[3]

Such interactions are occurring daily in each healthcare facility. It is a global healthcare issue, in which the patient involvement is required as a key.

It is important to note that the patient himself should be involved in this communication and decision-making process. Having said that, its practical applicability may be limited by gender, health status, education level, sociocultural beliefs, and the existing healthcare culture.[4,5]

With the prevalence of the aforementioned limitations, the point to make the patient involved seems to be achievable through any applicable channels of communication. The patient should be the key link in the communication process in the healthcare facility. An optimal way to tackle it would be to keep the patient at the center of communication, by way of ensuring the patient’s opinions receive prime attention.[6]

It would, therefore, be a good step in the healthcare field to create such a physician–relative–patient model and implement it. I found the old model shown in Figure 1 to be outstanding in illustrating the contact problem. It also explained effectively how many family members should be consulted or allowed to listen or give opinions about a patient’s management.

It was a good idea to draw the initial template to show the previous status. Further efforts are outstanding in designing a new one that involves finding the best way to communicate. The latter may simply be established, but as normal, the change process expected it to be challenging.

Additionally, creating a model that allows the patient to select only one “most responsible family member (MRFM)” may close the door of conflict when the need arises. Finally, this model will make it clear to all parties as to the individual who has been selected for delegatory decisions.

To sum up, I found the article to be of relevance to the Saudi healthcare context as it addresses the mechanism of interaction between patients, family members, and providers of health care. I write to the author(s) these few words because I would like to publish an analysis article to support it as a global strategy.
